# Supplementary material for: Alternative activation generates IL-10 producing type 2 innate lymphoid cells
Source: Nat Commun. 2017 Dec 1;8:1900. doi: 10.1038/s41467-017-02023-z (PMC5711851; doi:10.1038/s41467-017-02023-z)
Supplement: Supplementary file 1 — Supplementary Information [file 41467_2017_2023_MOESM1_ESM.pdf]

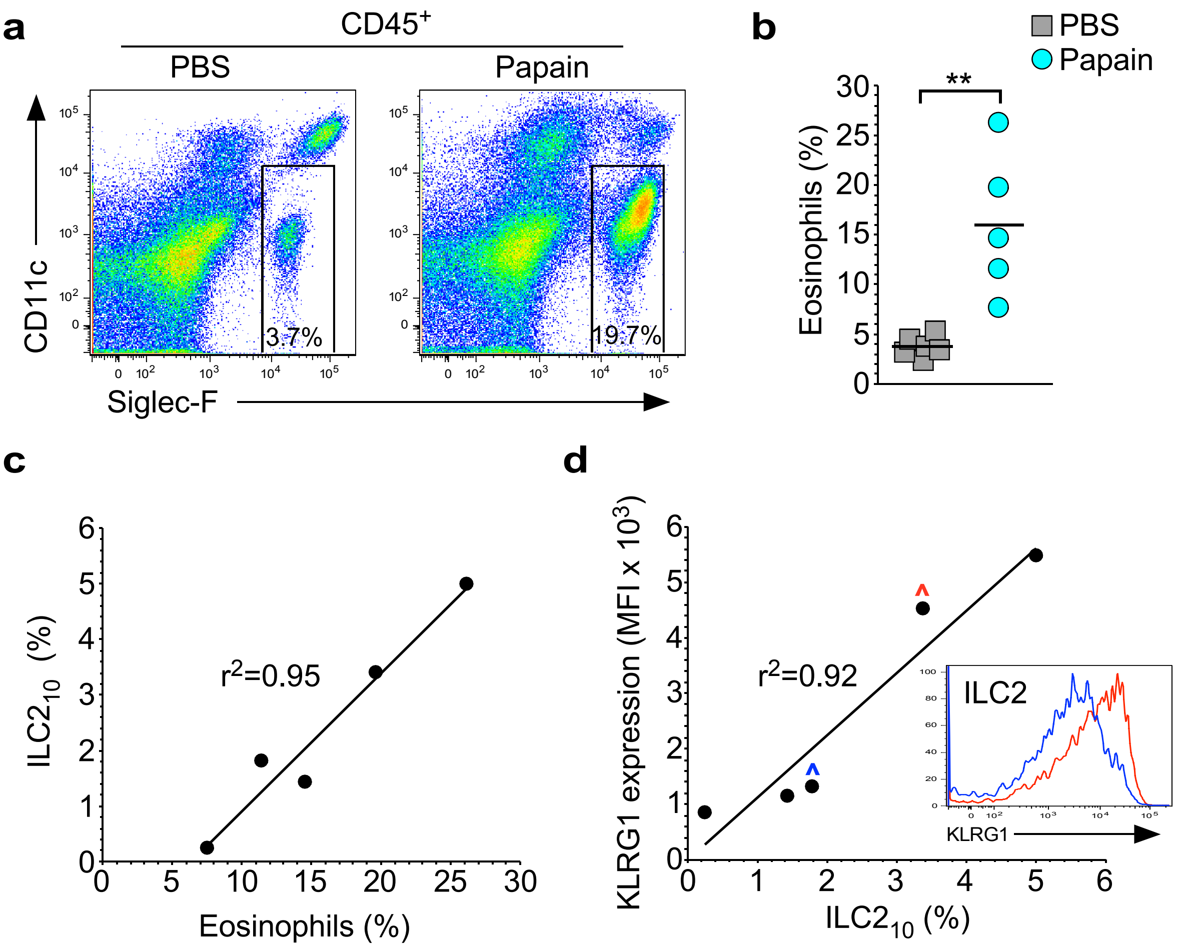

Supplementary Figure 1. Papain induces $ILC2_{10}$ production

(**a**) Analysis of lung eosinophils in 10BiT*Foxp3*^{GFP} reporter animals treated intranasally with papain. (**b**) Compiled data as in (**a**). (**c**) Comparison of $ILC2_{10}$ frequency with eosinophil frequency in papain treated mice. (**d**) Comparison of MFI of KLRG1 expression on total ILC2 with $ILC2_{10}$ frequency in papain treated mice. Inset histograms show KLRG1 expression on ILC2 from data points marked by corresponding colored carets. Data are from two independent experiments with representative plots shown in (**a**) and compiled data on individual mice shown in (**b,c,d**). **P < 0.01 (Student's t-test).

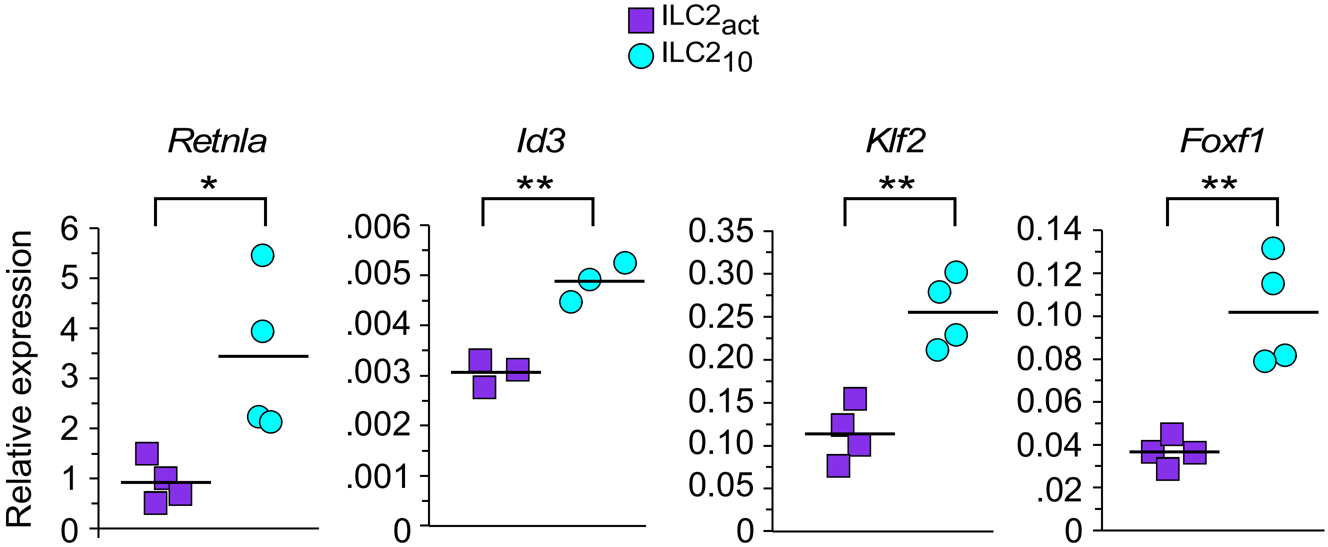

Supplementary Figure 2. Expression of select genes identified by RNA-seq

10BiT*Foxp3*^{GFP} dual reporter mice were treated with IL-33, and $ILC2_{act}$ and $ILC2_{10}$ purified by cell sorting. Shown is gene expression assessed by qRT-PCR. Each symbol represents data from an individual mouse and horizontal lines indicate the mean. These RNA samples were independent from those that were used to generate RNA-seq data. $^{**}P < 0.01$, $^{*}P < 0.05$ (Student's t-test).

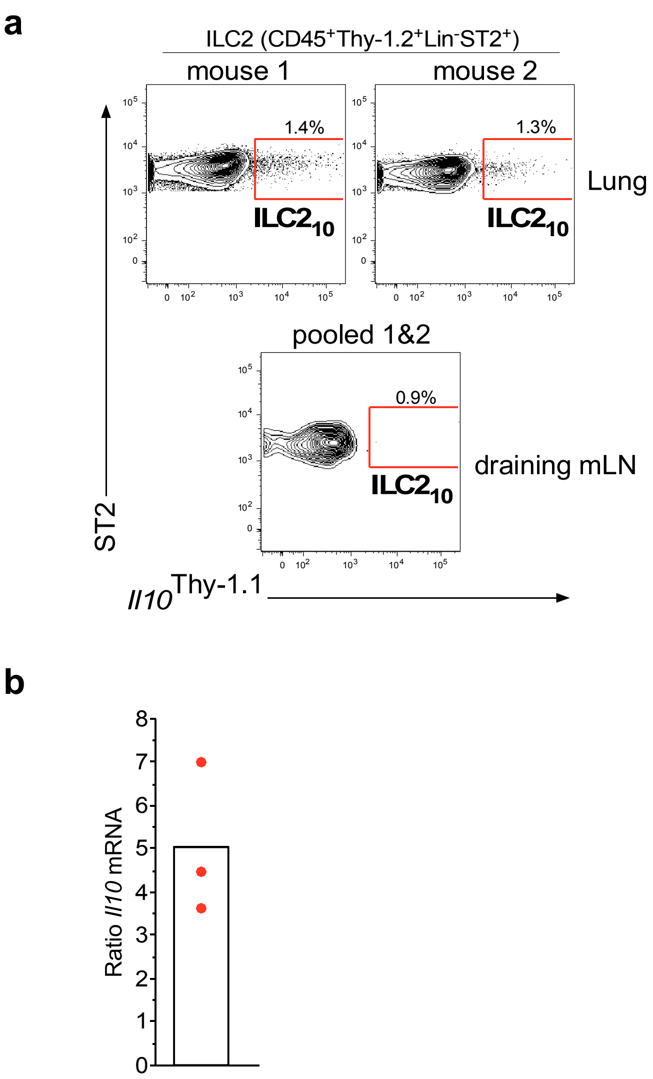

Supplementary Figure 3. Analysis of ILC2$_{10}$ at days 14 and 15 of recall experiments

(**a**) Staining for ILC2$_{10}$ in lung and draining mediastinal LN (mLN) in IL-33 injected mice at day 14 as in Fig. 4a. Lung ILC2 cells were analyzed in two individual mice, while mLN cells from these mice were pooled. (**b**) ILC2$_{10}$ and ILC$_{act}$ were isolated at day 15, following recall IL-33 administration as in Fig. 4a, and *Il10* gene expression determined by qRT-PCR. Shown is the ILC2$_{10}$:ILC2$_{act}$ ratio of *Il10* gene expression. Bar is average of three independent experiments pooling 2 mice per experiment with dots representing individual experiments.

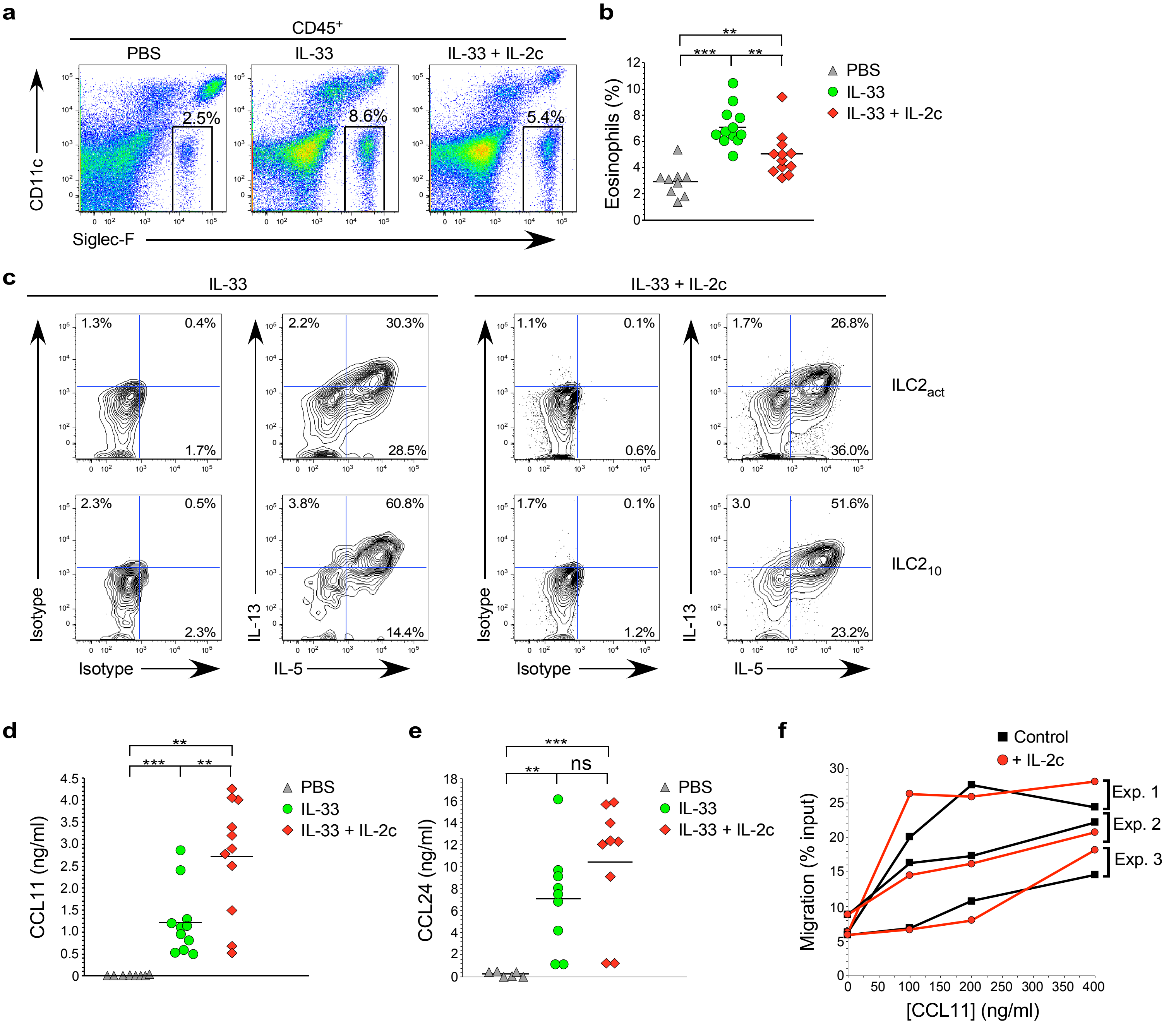

Supplementary Figure 4. ILC2$_{10}$ responses and inhibition of eosinophilia

(**a**) Wildtype mice were injected with PBS or the indicated cytokines for four consecutive days and analyzed for eosinophils the following day. (**b**) Compiled data of eosinophil frequency from mice treated as in (**a**). (**c**) Intracellular cytokine staining of PMA and ionomycin activated lung cells, derived from 10BiT reporter mice treated with IL-33 or IL-33 + IL-2c, gated on ILC2$_{act}$ (CD45$^+$Thy-1.2$^+$Lin$^-$ST2$^+$Thy-1.1$^-$) or ILC2$_{10}$ (CD45$^+$Thy-1.2$^+$Lin$^-$ST2$^+$Thy-1.1$^+$) cells. (**d,e**) Determination of lung CCL11 (**d**) or CCL24 (**e**) in mice treated as indicated. (**f**) Migration of splenic eosinophils in culture to CCL11 in the presence or absence of IL-2c. Results of 3 independent experiments are shown, with each point on the graph representing the average of duplicate wells. Other data are from four (**a,b,d**), three (**e**) or two (**c**) independent experiments with representative plots shown in (**a,c**) and compiled data on individual mice shown in (**b,d,e**). ***P < 0.001, **P < 0.01, and ns (not significant, P $\geq$ 0.05) (Students t-test).

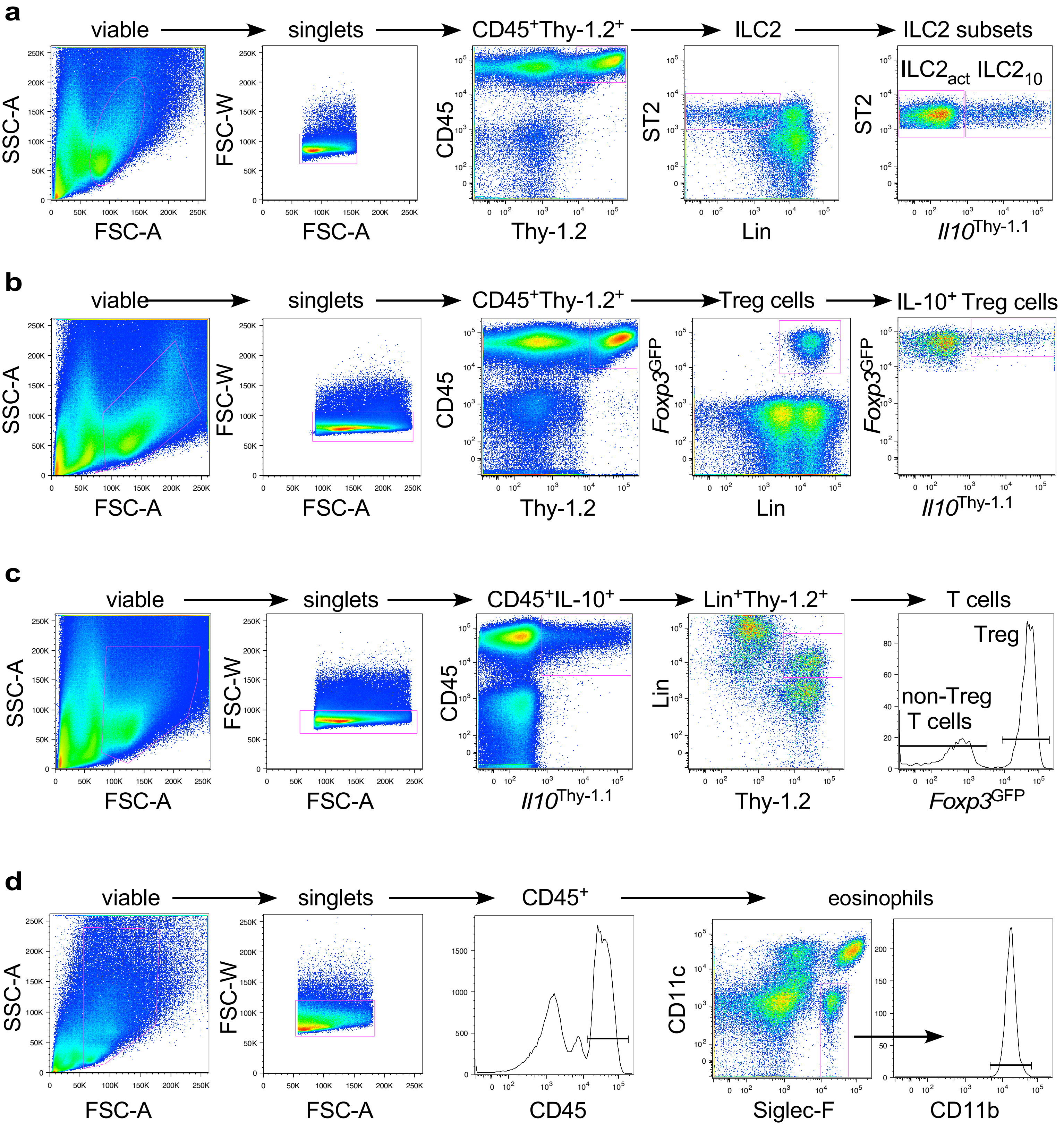

Supplementary Figure 5. Flow cytometry gating strategy for lung cells

(**a**) Gating for ILC2, $ILC2_{act}$, and $ILC2_{10}$ used throughout the manuscript. (**b**) Gating strategy used for IL-10^+ Treg cells, as in Fig. 2b,c,d. (**c**) Gating strategy used for Treg cells and non-Treg T cells within the IL-10^+ cell population, as in Fig. 2f. (**d**) Gating strategy used for eosinophils in Fig. 7 and Supplementary Figs. 1a and 4a.
