# Supplementary material for: Alternative activation generates IL-10 producing type 2 innate lymphoid cells
Source: Nat Commun. 2017 Dec 1;8:1900. doi: 10.1038/s41467-017-02023-z (PMC5711851; doi:10.1038/s41467-017-02023-z)
Supplement: Supplementary file 3 — Description of Additional Supplementary Files [file 41467_2017_2023_MOESM3_ESM.pdf]

Description of Additional Supplementary Files

File Name: Supplementary Data 1
Description: Differentially expressed genes upon ILC2 activation

File Name: Supplementary Data 2
Description: Differentially expressed genes comparing $ILC2_{act}$ and $ILC2_{10}$

File Name: Supplementary Data 3
Description: RNAseq dataset from cultured ILC2
